# Trends in Intelligent and AI-Based Software Engineering Processes: A Deep Learning-Based Software Process Model Recommendation Method

**DOI:** 10.1155/2022/1960684

**Published:** 2022-10-05

**Authors:** Fahad H. Alshammari

**Affiliations:** College of Computing and Information Technology, Shaqra University, Shaqra, Saudi Arabia

## Abstract

In recent years, numerous studies have successfully implemented machine learning strategies in a wide range of application areas. Therefore, several different deep learning models exist, each one tailored to a certain software task. Using deep learning models provides numerous advantages for the software development industry. Testing and maintaining software is a critical concern today. Software engineers have many responsibilities while developing a software system, including coding, testing, and delivering the software to users *via* the cloud. From this list, it is easy to see that each task calls for extensive organization and preparation, as well as access to a variety of resources. A developer may consult other code repositories, websites with related programming content, and even colleagues for information before attempting to build and test a solution to the problem at hand. In this investigation, we aim to identify the factors that led to developing the recommender. This system analyzes the recommender's performance and provides suggestions for improving the software based on users' opinions.

## 1. Introduction

When developing a software system, software engineers execute various tasks, including creating code, testing code, deploying to the cloud, and coordinating *via* e-mail and meetings [1]. Each of these tasks necessitates searching for and working with a wide range of information and resources, as well as planning and preparing for the upcoming one [2]. A developer may investigate other code repositories for prospective solutions, explore online sites with relevant programming material, or contact coworkers for information before programming a possible solution to the problem at hand and testing the answer [3].

For example, performing these tasks can be intimidating for novices in the field [4]. Near-perfect performance in these activities is nearly unattainable for even the most experienced coders. Recommender systems for software engineering have been implemented to easily perform tasks and improve workflow [5]. In other words, “software applications that deliver information items deemed to be relevant for software engineering tasks” are “recommenders” for the discipline [6]. Software engineers are used to working with certain recommenders that are closely relevant to their development operations. Such issues as missing import declarations in Java code can be solved using recommenders in various integrated development environments, such as the Eclipse IDE4 [7]”.

Recommendation systems for different tasks and workflows have been developed, including those for code reorganization, learning the next set of commands, and discovering needs. For instance, the Eclipse Mylyn recommender, which provides specific recommendations of which source code is connected with a task, has been demonstrated to boost the productivity of developers. Recommenders have much unrealized potential in the software development process because of their vast variety of actions [8].

One of the primary problems with the current recommender system is that it forecasts products that the user will find irrelevant or uninteresting. As a result, a recommender system is required, which must supply services in accordance with the resemblance of goods. By incorporating user and product data into a collaborative recommendation system, true user preferences can be learned [9–11].

The first stage in developing a recommender is to define the problem the recommender is intended to solve and verify the assumption that a recommender can deliver suggestions of value to the developer facing the problem. Framing the problem is the term we use to describe the activities occurring during this phase. The introduction's definition of a software engineering recommender provides a foundation for investigating the problem and solution targeted by a recommendation engine. The task and context for which a recommender will be used must be crystal apparent when thinking about creating one. Another consideration is for whom a recommender is intended: developers or end users. The idea of a task targeted by a recommender relates to the specific purpose of a developer at a certain moment in time, such as the implementation of an assigned feature in source code. Even though a developer is aware of the current task at all times, the task may not be expressed directly in the code. The context of a recommender refers to the information and tool environment in which the task is conducted, such as the source code and other artifacts available and the set of tools that can be used to complete the work. The context also captures the developer's steps in completing the given task. This helps define when and what information a recommender may provide: novices often have fundamentally different information needs compared to experts. While frequent proposals may be helpful to the first group, the latter often has a poor tolerance for interruptions of their work that convey already known facts. The main contributions of this study are as follows:

- Determining the inputs for the recommender's construction was how we phrased the issue
- This system provides recommendations for software development based on client happiness and evaluates the usefulness of the recommender

## 2. Related Work

Wen et al. [12] systematically examined machine learning models from four perspectives: the kind of ML approach, estimation accuracy, model comparison, and context of estimation, which is the goal of this study. A systematic review of empirical studies on the ML model published between 1991 and 2010 was conducted. The author compiled a list of 84 primary studies related to our research question and detected eight different types of ML approaches used in SDEE models after looking into these studies. Overall, these ML models have better estimation accuracy than non-ML models and are near to it. For this reason, certain ML models are more effective in certain estimation scenarios. SDEE is a potential field for ML models. However, the industry's use of ML models is still limited, necessitating additional efforts and financial incentives. Following the conclusions of this review, the author offers advice for researchers and guidance for practitioners.

Wan et al. [13] were curious about the impact of machine learning on software development techniques, given the growing popularity of this approach. From interviews with 14 people and surveys with 342 people from 26 nations across four continents, we could identify substantial differences between the development of machine and nonmachine learning systems. Software engineering (e.g., requirements, design, testing, and process) and work characteristics are significantly different across the two groups, according to our research (e.g., skill variety, problem-solving, and task identity). In light of our findings, the author outlined potential future research areas and offered practice-oriented suggestions.

Del Carpio and Angarita [14] used machine learning approaches in various knowledge domains with promising results. Many deep learning models now focus on a wide range of software operations, which is a good sign for the future systematic investigation of deep learning model-supported software processes that yield useful findings for the software industry. Software testing and maintenance were the most often studied subprocesses in this study. It is common to utilize deep learning models such as CNN and RNN to process bug reports, malware categorization, and recommendation creation in these subprocesses. Some solutions are focused on estimating effort, classifying software requirements, identifying GUI visual aspects, identifying code authors, finding the similarity between source codes, predicting and classifying defects, and analyzing bug reports in testing and maintenance operations.

Meziane and Vadera [15] suggested that, due to its ability to automate time-consuming or complex processes, artificial intelligence has recently gained much attention. There have been no exceptions to this rule regarding software engineering projects. Artificial intelligence and software maintenance are covered in depth in this thesis. The recent advances in applying artificial intelligence to software maintenance duties were also studied through thorough mapping research. Research kind, research contribution, software maintenance domains, and artificial intelligence solution type were the most important aspects of this study.

Barenkamp et al. [16] involved a systematic evaluation of prior research and five qualitative interviews with software developers. The study's conclusions are categorized throughout software development. Major AI achievements and future potentials include (a) using algorithms to automate time-consuming, routine tasks in software development and testing (such as bug hunting and documentation); (b) conducting structured analyses of large datasets to uncover patterns and new information clusters; and (c) conducting systematic evaluations of these datasets in neural networks. AI accelerates development, reduces expenses, and increases efficiency. Software engineering automation is superior to the present AI, which relies on human-made structures and is essentially reproductive. Developers can enhance their creativity with AI tools.

Harman claimed that the artificial intelligence (AI) approaches to software engineering also focuses on the software development related challenges [17]. While search-based software engineering is a more recent development, the field's history of work in probabilistic reasoning and machine learning for software engineering is well-established. For the purpose of this paper, the author examined some of the connections between these two areas of research, claiming that they share many characteristics.

Tate [18] compared software quality models. Case studies apply software quality models to the current processes. Case study results complement empirical model assessment. Standard selection criteria are used to recommend and select models. Procedures are evaluated using success criteria. Theoretical assessment methods evaluate process model quality. Conformity to ideal process quality model requirements and relevance to software stakeholders are tested. Discussing the models' breadth and scale: empirical assessment methods are established to evaluate the model's performance in real software operations. There are approaches to determine if process quality models produce different results and, if so, which model to choose. Case study software processes are measured for differences.

Fadhil et al. [11] determined how AI can improve software issue detection and prediction methods. Artificial intelligence has helped identify software issues and predict bugs, as data shows. Combining AI with software engineering reduces overhead and produces more efficient solutions, improving software quality.

Kothawar and Vajrapu [19] addressed these behaviors' difficulties and solutions. Methods: the author chose 15 best practices from eight startups, each with unique challenges and solutions. Our research indicates startups' mixed prioritization. Six of the eight companies used formal methods, while two used unstructured prioritization. Startups' value: prioritizing based on consumer input and ROI is key. This study examines startup priority needs and obstacles. The literature supports the study's findings. Finding solutions helps practitioners. The poll should include Swedish software startups. Some of these solutions may also be useful for practitioners wishing to begin a software startup and priority requirements.

This study's aggregation method is clear, realistic, and interpretable [9]. This method makes quality model and metric-based software quality assessment reliable and reproducible. Based on all observable software artifacts, good and bad quality are assigned probabilities. Validation was theoretical and empirical. Bug prediction, maintainability, and information quality were evaluated. Software visualization was used to evaluate the usefulness of aggregation for multivariate data and the impact of different aggregation methods. Finally, the author assessed MCR's transferability and used it to rate real-world options. The author used machine learning, created a benchmark employing regression issues, and evaluated how well the aggregate result matches a ground truth and represents input variables. Our method is accurate, sensitive, and facilitates multicriteria decision-making. Our approach can be used as an agnostic unsupervised predictor without ground truth.

Recently, sentiment analysis on social networks, such as Twitter and Facebook, has become a valuable tool for gaining insight into the thoughts and feelings of people. In contrast, sentiment analysis suffers from the difficulties of natural language processing (NLP). Deep learning models have recently been a promising solution to NLP difficulties. To address the issues with sentiment analysis, such as sentiment polarity, the paper [10] analyzes the most recent experiments to make use of deep learning. Word embedding and the TF-IDF model have been used to analyze several different datasets. Comparative studies of the experimental findings for various models and input features have also been undertaken.

Software defect prediction anticipates troublesome code sections to help find faults and priorities testing. Previous work focused on manually encoding program information and using machine learning to generate accurate prediction models. Standard characteristics do not capture semantic differences between programs for accurate prediction models [8]. Deep learning is proposed to bridge the gap between program semantics and fault prediction characteristics. The deep belief network (DBN) learns semantic features from Abstract Syntax Tree (AST) token vectors automatically. Our research on 10 open-source projects shows that our automatically learned semantic features increase both within-project and cross-project defect prediction over traditional characteristics. Precision, recall, and F1 improve WPDP by 14.7%, 11.5%, and 14.2%, respectively. Our semantic feature-based technique beats TCA + by 8.9% in F1 for CPDP.

Reference [20] proposed LEMNA, a high-fidelity security explanation approach. LEMNA generates a limited set of features that explain how an input sample is categorized. The goal is to create a simple interpretable model to approximate the deep learning decision boundary. It manages feature dependency to better interact with security applications (such as binary code analysis) and nonlinear local boundaries to boost explanation fidelity. Local interpretable model (LIM): the author tested our method with two deep learning security apps (a malware classifier and a function start detector for binary reverse engineering). Extensive testing demonstrates that LEMNA's explanation is more correct than others. The author shows how LEMNA may help machine learning developers verify model behavior, fix classification issues, and automatically patch target model defects.

Reference [7] reviewed machine learning papers for software project management. Web Science, Science Directs, and IEEE Explore have research on machine learning, software project management, and methodology. Three repositories contain 111 papers in four groupings. First group: software project management papers. The second category contains machine learning methods and tactics utilized in projects. The third category comprises studies on machine learning management phases and tests, as well as study findings, contribution to and promotion of machine learning project prediction, and other studies. It gives a broader context for future project risk management efforts. Machine learning-based project risk assessment is more successful in reducing project losses, increasing project success, and reducing project failure probabilities while increasing the growth output ratio.

Recent machine learning discoveries have prompted interest in integrating AI into IT software and services. To fulfill this goal, organizations adapted their development methodologies. The author shares research on Microsoft's AI-app development teams. It is built on designing AI apps (search and NLP) using data science tools (R and Python) (e.g., application diagnostics and bug reporting). Reference [5] found that multiple Microsoft teams have integrated this workflow into established, well-evolved software engineering processes, providing insights into numerous important engineering problems organizations may encounter while developing large-scale AI products for the market. These difficulties required Microsoft's best practices. Aside from that, the author found three main AI differences: (1) model customization and reuse demand different abilities than those found in software teams. (2) AI components are more challenging to handle as independent modules than typical software components. Microsoft teams provided critical knowledge.

Yang et al. [6] proposed “deep neural networks” (DNNs) and an updated model training approach. Alpha Go showed deep learning's potential in 2016. Deep learning helps software engineering (SE) experts construct cutting-edge research tools. Model selection, internal structure, and tuning affect DNN performance in SE. Deep learning in SE is understudied. The author searched for relevant publications since 2006. First, SE deep learning is shown. SE's deep learning methods are classified. The author looked at deep learning model optimization methodologies and highlighted SE research problems that will benefit from DNNs. Our findings highlight existing problems and suggest a potential study route.

Machine learning is rapidly used by the software engineering community as a means of transforming modern software into intelligent and self-learning systems. Software engineers are still exploring methods in which machine learning can aid with various stages of the software development life cycle. Herein, the author reports the results of a study on the application of machine learning at various stages of the software development life cycle. Overall, [3] investigated the relationship between software development life cycle stages and machine learning tools, techniques, or types, which is a broad goal. In an attempt to answer the question of whether machine learning favors specific stages or methodologies, we conduct a comprehensive analysis.

Business transactions, revenues, and general success are becoming increasingly dependent on the use of recommendation systems. Recommendation systems and their implementation approaches are the focus of this survey. The components and attributes of a recommender system can change based on the organization's needs. Design criteria and key recommender system attributes are presented in this study. There are a few well-known approaches that are scrutinized. In conclusion, [4] introduced movie recommenders from the three most relevant industries: film, music, and online shopping. The survey seeks to provide readers with a broad understanding of the circumstances in which certain recommender systems are appropriate.

Machine learning models are frequently developed by data scientists to handle a wide range of problems in both industry and academia, but they are not without their own set of hurdles. One of the issues with machine learning development is that many people working in the field are unaware of the benefits that may be reaped from following the steps outlined in the software engineering development lifecycle (SEDL). Of course, because machine learning systems are distinct from typical software systems, there will be certain peculiarities in the development process. Regarding software engineering, [2] aimed to examine the issues and practices that arise during model creation by looking at how developers might benefit from using or changing the standard workflow to machine learning.

Software engineering has recently used deep learning (SE). Unanswered questions remain. Li et al. [1] looked at 98 SE publications that employ deep learning to tackle these questions. Deep learning technologies have simplified 41 SE jobs across all phases. Deep learning models and their variations are utilized to answer 84.7% of SE issues in publications. Deep learning's practicality is questioned. More SE scholars may be interested in improving deep learning-based solutions in the future.

## 3. Methodology

**Figure 1:**
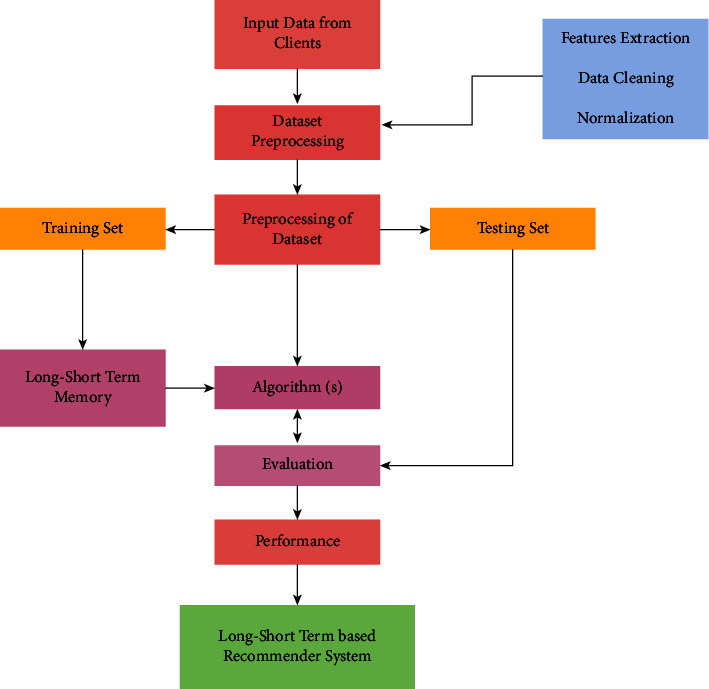
The proposed framework workflow.

In this section, we have proposed a novel framework of LSTM which can recommend the software development features based on the dataset of clients.  Figure 1 shows the proposed framework workflow of the current study:

### 3.1. Dataset Description

The dataset used in this study is an excel-generated synthetic dataset curated from a real BI tools' dataset. This dataset has 100 rows and 11 features with 1 output feature (i.e., rating); when the rating of software is more than 3, this will be recommended. Otherwise, it will not be recommended by the proposed model.  Table 1 shows the dataset description and feature explanation.

Table 2 shows the dataset samples from the acquired dataset as given below.

**Figure 2:**
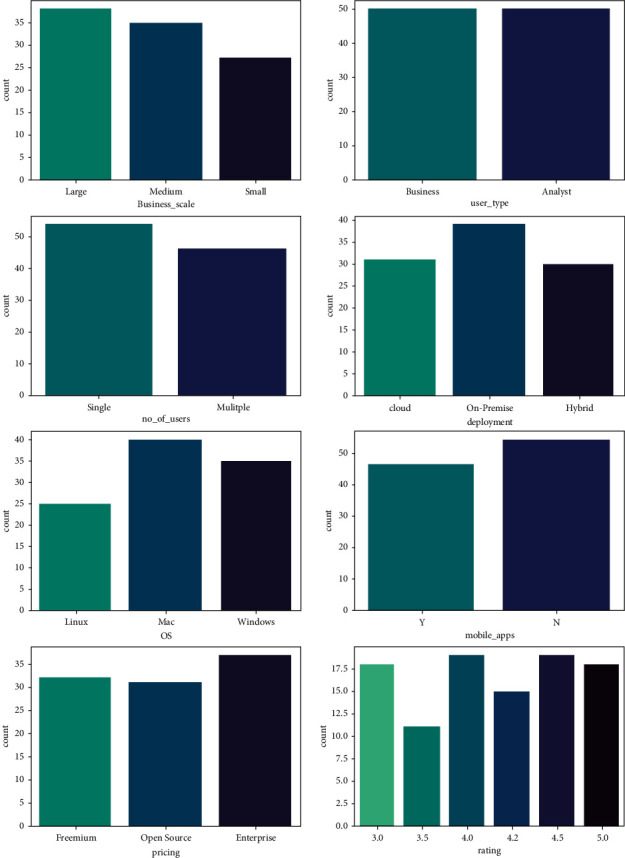
Visualization of the dataset and frequency distribution of attributes.

Figure 2 shows the visualization of the dataset and frequency distribution of each feature as given below.

**Figure 3:**
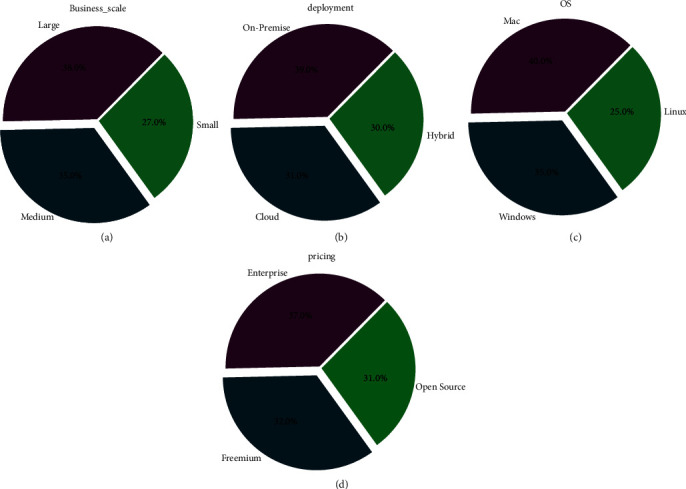
Distribution of features: (a) business scale, (b) deployment, (c) OS, and (d) pricing.

Figure 3 shows the distribution of feature business scale with respect to large, small, and medium deployment on premise, hybrid, and cloud OS for Windows, Mac, and Linux and pricing on Freemium, open source, and enterprise.

### 3.2. Raw Data Processing

The raw data have been collected. Finally, data purification has been completed using various methods, such as deleting duplicates and null values. This technique is employed in data mining to transform unstructured data into a form suitable for analysis. It is not uncommon for data in the real world to be inconsistent or even missing. Prediction models are complicated when classifications are not dispersed uniformly throughout. The number of occurrences in each class is often the same in categorization machine learning algorithms. In the wake of this study, resampling procedures have substantially evolved. Remove records from each cluster such that the majority class records are captured and undersampling is prevented. For more diverse synthetic samples, oversampling can be utilized in place of producing identical reproductions of data from the minority classes [21]. When conducting data mining research, it is critical that our dataset is balanced and consistent. It is possible to find outliers in a dataset. An outlier in a dataset is a value that stands out from the rest because of its uniqueness. The outliers could result from reading errors, equipment faults, or human error. Before undertaking any statistical analysis or study, it must be deleted from the dataset. The analysis and subsequent treatment can be influenced by incomplete or erroneous findings from any information outliner [22, 23].

### 3.3. Feature Engineering

By using data from a certain domain, learning machines can use these functions. In order to make machine learning representations of raw data, this must be done manually. Correlation matrices are used in this study to determine the correlation between the variables. Covariance matrices are the same as correlation matrices. Using the correlation, one may determine the strength of a linear link. The concept of correlation summarizes the frequency and direction of a straight-line link between two quantitative variables. Values can be represented by *r*, which ranges from −1 to +1.

### 3.4. Proposed Model

**Figure 4:**
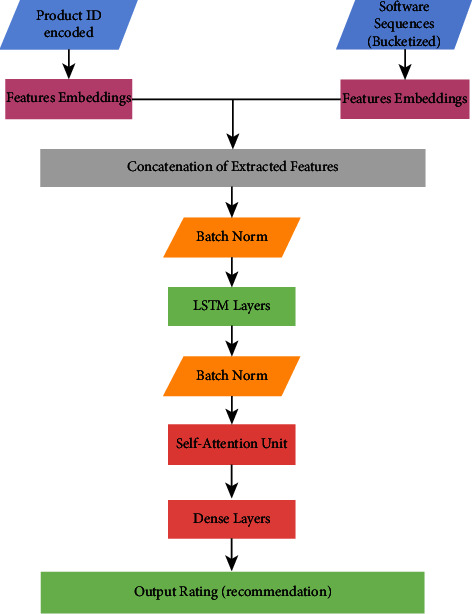
Proposed model architecture.

In the proposed model (shown in Figure 4), input sequences are feature embedded and then extracted in the contented layer. There is a hyperband optimization algorithm that can be used to distribute hyperparameter tuning for TensorFlow models in just a few lines of code in the Keras–Tuner module. For hyperparameter tuning, a validation dataset containing 10% of randomly selected samples from the training data is used. Furthermore, we employed sparse categorical accuracy as a ranking metric for optimization trials. We experimented with various batch size variables before settling on batch size = 512. Data from previous optimization stages are used to train a final model with a set of hyperparameters that is as good as it can possibly be. In order to assess the accuracy of our new recommender system, we implemented a back-testing technique.

#### 3.4.1. Novel LSTM Cell

**Figure 5:**
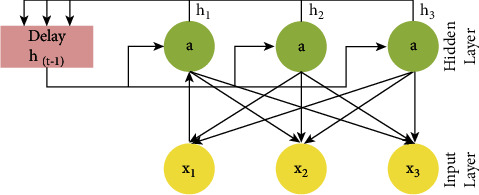
Recurrent nodes of modified LSTM.

**Figure 6:**
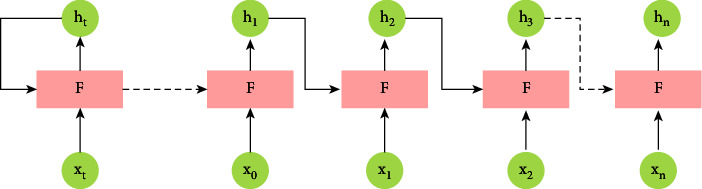
Unrolled nodes.

Long short-term memory networks are a subset of the broader category of recurrent neural networks. An example of time- or sequence-dependent behavior is language, stock prices, and power demand; recurrent neural networks seek to represent such phenomena. In order to achieve this, the output of a layer in a neural network at time *t* is fed back into the input of the same layer at time *t* + 1.  Figure 5 shows the modified recurrent units of the new version of LSTM:

During training and prediction, recurrent neural networks are “unrolled” programmatically, resulting in Figure 6.

New data are sent to the network at each time step, and the output of the previous *F* ($h *t*−1$) is also supplied, as shown in Figure 6.

**Figure 7:**
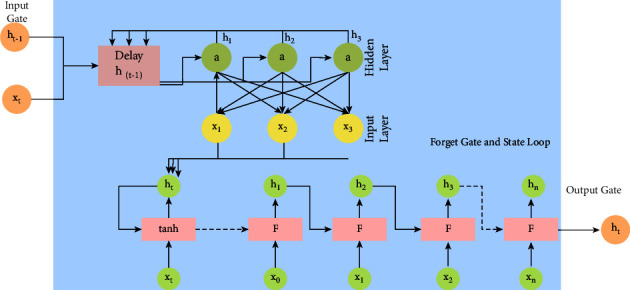
Modified cell of LSTM.

In place of the typical neural network layers, an LSTM network uses LSTM cell blocks to store information for future use. The input, forget, and output gates are all parts of these cells that will be discussed in greater depth below. Our planned LSTM cell is depicted graphically below in Figure 7.

#### 3.4.2. Input Gate

First, a tanh activation function is applied, compressing the input to a range from −1 to 1. To put it another way,

(1) $$\begin{array}{c} g=\tanh\left(b^{g}+x_{t}^{U^{g}}+\;h_{t-1}V^{g}\right), \end{array}$$

where *x*_*t*_^*U*^*g*^^ and *V*^*g*^ represent the input and previous cell output weights and *b*^*g*^ represents the input bias. The *g* exponents do not represent an increased power but rather the weights and biases used in the input calculations (as opposed to the input gate, forget gate, output gate etc.). The output of the input gate, which is a chain of sigmoid-activated nodes, is multiplied by this compressed input, element by element:

(2) $$\begin{array}{c} i=a\left(\left(b^{i}+x_{t}^{U^{i}}+\;h_{t-1}V^{i}\right)\right). \end{array}$$

#### 3.4.3. Forget Gate and State Loop

Forget gate of the cell is expressed as

(3) $$\begin{array}{c} f=a\left(\left(b^{f}+x_{t}^{U^{f}}+\;h_{t-1}V^{f}\right)\right). \end{array}$$

The product of the previous state with the forget gate yields an expression of the form ((*b*^*f*^+*x*_*t*_^*U*^*f*^^+ *h*_*t*−1_*V*^*f*^)) as its output. Following the forget gate/state loop, the product is

(4) $$\begin{array}{c} s_{t}=\;s_{t-1}x\;f\;x\;g. \end{array}$$

#### 3.4.4. Output Gate

The output gate of LSTM is expressed as

(5) $$\begin{array}{c} O=a\left(\left(b^{o}+x_{t}^{U^{o}}+\;h_{t-1}V^{o}\right)\right). \end{array}$$

Finally, the product of all gates is

(6) $$\begin{array}{c} h_{i}=\;\tanh\left(a\left(\left(b^{o+f+s+i}+x_{t}^{U^{o+f+s+i}}+\;h_{t-1}V^{o+f+s+i}\right)\right)\right). \end{array}$$

Recall and accuracy were utilized to assess the effectiveness of the strategies under consideration for the software development recommender system. The computations of the metrics utilized in this study are shown in Table 3.

## 4. Results and Discussion

Our approach was put to the test using data from the Steam project. In order to test our strategy, there are no existing datasets that can be used for this purpose. For testing purposes, we used the most recent records as a test set and the rest of the records as training sets.

In this experiment, we used a serial filling with a time series length of *T* = 12 and a dimensionality reduction with an aimed dimension *k* = 50. Finally, we gave each user a list of the top 50 (*N* = 50) things. We used two separate control trials to assess the effectiveness of each component of our strategy. Neither the serial filling (noSF) nor the dimensionality reduction (noDR) was applied in one experiment.

In order to establish a baseline, we compared our method to collaborative filtering for implicit feedback (IF) and temporal decay (TD). To evaluate the correctness of our recommendations, we looked at the recall rate, whereas for determining system efficiency, we looked at training and execution times. Our final step was to examine each software's average recommendation time to see if there was a wide range of recommendation times for each method.

The recall rates for various techniques are shown in Table 4. When IR and serial filling were tested, it was found to have a greater recall rate than the baseline techniques. Time spent in IR is shown in Table 5. Matrix factorization is a useful way to reduce the number of dimensions in a system because the recall rate of IR was nearly the same as that of IRnoDR.

**Figure 8:**
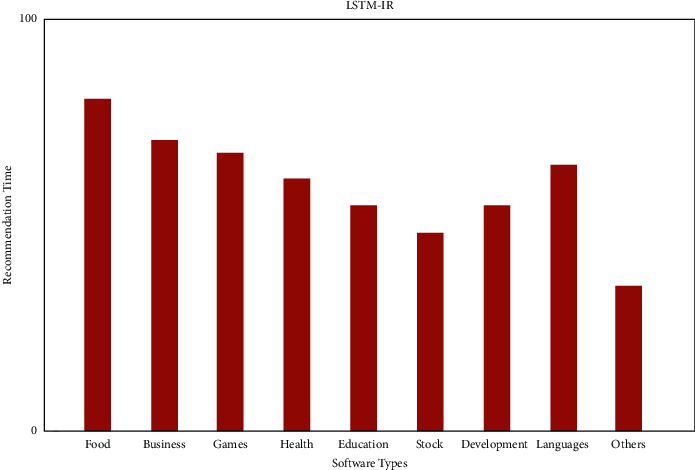
Recommendation of LSTM with IR.

**Figure 9:**
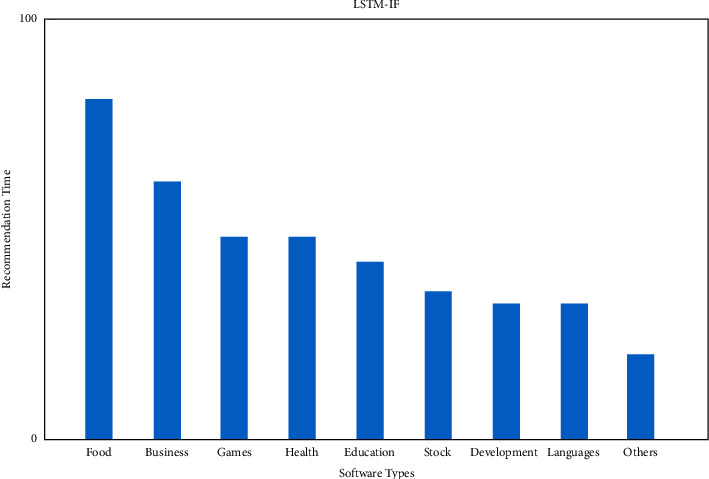
Recommendation of LSTM with IF.

Figure 8 displays the IR and Figure 9 depicts the IF distribution of the top software recommendation times. Because IR recommends more diverse items than baseline collaborative filtering, we can conclude that our approach is more diverse than baseline collaborative filtering.

## 5. Conclusions

Within the scope of this work, an LSTM-based recommendation model for interaction records was suggested. Based on the results of our evaluations, our model performed admirably in all three categories: accuracy, efficiency, and variety. In the future, we intend to evaluate the generalizability of our approach by applying it to a wide variety of datasets. In addition, we considered the total amount of time spent communicating with one another as a quality factor in this study. There is a high probability that reviews will be distorted due to the viewpoints of various individuals and types of goods. As a direct consequence of this, we ought to direct our attention going forward toward enhancing the quality of our rating vectors in the future. In order to deal with time series, we will also investigate a variety of other approaches and models.

## Figures and Tables

**Table 1:** Dataset description and feature explanation.

Features	Description	Variables type
Category	Category comprises the type of BI tool, as well as the industry in which it can be used	Input variable
Business scale	This identifies the size of the company that the BI tool is designed to serve, such as small, medium, or large	Input variable
User type	Whether they are a business user or an analyst with data science skills, this indicates the sort of user (not all BI tools are easy to use and not all tools possess powerful data processing capabilities)	Input variable
No of users	This offers information on how the BI tool may be implemented, such as cloud, on-premise, or hybrid OS: this specifies the type of operating system necessary for the tool installation	Input variable
Pricing	Pricing: this reveals whether the program has a freemium version or an enterprise edition for data visualization on mobile devices	Input variable
Ratings	On a scale of 5.0, users rate this product	Output variable

**Table 2:** Dataset samples from the acquired dataset.

Category	Industry	Business scale	User type	No. of users	Deployment	OS	Mobile apps	Pricing	Rating
100001	Data management	Utilities	Large	Business	Single	Cloud	Linux	Y	4.5
100002	Database/ERP	Food	Large	Business	Single	Premise	Mac	Y	5.0
100003	Data analysis	Manufacturing	Large	Business	Single	Premise	Linux	N	5.0
100004	Data analysis	IT	Medium	Business	Multiple	Premise	Mac	Y	4.3
100005	Benchmarking	Food	Medium	Analyst	Multiple	Cloud	WIN	N	4.7

**Table 3:** Description of metrics.

Metric	Description
Accuracy	Accuracy =*TP*/(*TP*+*TN*)*∗*100
Recall	Recall =∑recommender/∑(new − known)*∗*100

**Table 4:** Recall rates of the proposed LSTM with different approaches.

Approach	Recall rate
IR	0.0834
IF	0.054
TD	0.012

**Table 5:** Time of the proposed LSTM with different approaches.

Approach	Training time (seconds)	Testing time (seconds)
IR	1400	15
IF	1150	12

## Data Availability

The data used to support the findings of this study are included within this article.
